# HSV-1 DNA Replication—Coordinated Regulation by Viral and Cellular Factors

**DOI:** 10.3390/v13102015

**Published:** 2021-10-07

**Authors:** Jessica E. Packard, Jill A. Dembowski

**Affiliations:** Department of Biological Sciences, Duquesne University, Pittsburgh, PA 15282, USA; packardj@duq.edu

**Keywords:** herpes simplex virus, HSV-1, DNA replication, replication fork, recombination

## Abstract

DNA replication is an integral step in the herpes simplex virus type 1 (HSV-1) life cycle that is coordinated with the cellular DNA damage response, repair and recombination of the viral genome, and viral gene transcription. HSV-1 encodes its own DNA replication machinery, including an origin binding protein (UL9), single-stranded DNA binding protein (ICP8), DNA polymerase (UL30), processivity factor (UL42), and a helicase/primase complex (UL5/UL8/UL52). In addition, HSV-1 utilizes a combination of accessory viral and cellular factors to coordinate viral DNA replication with other viral and cellular processes. The purpose of this review is to outline the roles of viral and cellular proteins in HSV-1 DNA replication and replication-coupled processes, and to highlight how HSV-1 may modify and adapt cellular proteins to facilitate productive infection.

## 1. Introduction

Herpesviruses are a relatively large and diverse group of double-stranded DNA viruses that replicate in the nucleus of host cells. There are several herpesviruses that are known to infect humans, including HSV-1, HSV-2, varicella zoster virus, Epstein–Barr virus, Kaposi’s sarcoma-associated herpesvirus, cytomegalovirus, human herpes virus 6A (HHV6A), HHV6B, and HHV7. It is estimated that about 70% of the world population is infected with HSV-1 [1,2]. HSV-1 infections can result in blisters on the skin, mouth, lips, eyes, and genitals [3]. Infection can also come with increased severity, including herpes keratitis and encephalitis, especially in immunocompromised individuals and neonates [4].

Much of the HSV-1 infection cycle takes place within the nucleus of epithelial cells [5]. It is within the nucleus that viral and cellular factors interact with the 152 kbp HSV-1 genome to regulate viral DNA replication, transcription of viral genes, and viral genome packaging into capsids. The HSV-1 genome consists of over 80 genes, including those that code for mRNAs and non-coding RNA (ncRNAs) [6,7]. Viral genes are transcribed by cellular RNA polymerase II (RNA pol II) [8] through a temporal cascade of immediate early (IE or α), early (E or β), and late (L or γ) viral genes [9,10]. After primary infection of epithelial cells, HSV-1 establishes latent infection in sensory neurons for the lifetime of the host [11]. By unknown mechanisms, HSV-1 can re-enter the lytic infectious cycle following stress or other similar stimuli [12].

During lytic infection, replication of the viral DNA is catalyzed by seven core replication proteins, all of which are functionally analogous to the eukaryotic replication machinery [13]. In addition to viral factors, several cellular factors have been identified to be important for HSV-1 DNA replication and are enriched on viral replication forks [14,15,16]. This review will primarily focus on the mechanisms behind DNA replication of HSV-1 and the potential functions of viral replication fork-associated cellular factors.

## 2. Viral DNA Replication

### 2.1. Viral Genome Structure

Importantly for the mechanisms of viral DNA replication, the incoming HSV-1 genome enters the nucleus in a linear form. In the absence of ICP0 and during latency, the genome circularizes [17]. It is commonly thought that HSV-1 genomes also become circular during lytic infection. However, no circular genomes can be detected during lytic infection in the presence of ICP0 [17]. Although increased joint regions are observed during productive HSV-1 infection, these may be the result of concatemer formation, branching that occurs during DNA replication, or DNA recombination. In support of this, HSV replication intermediates include linear head-to-tail concatemers and branched structures [18,19]. The exact mechanism by which concatemers form is not known. In addition, replicating and encapsidated HSV-1 genomes contain single-strand breaks (SSBs) and gaps on both DNA strands [20]. How nicks and gaps contribute to the DNA damage response (DDR) at early and late stages of infection, as well as transcription, DNA replication, and recombination are not well understood.

During latency, there is clear evidence that the viral genome is associated with nucleosomes resembling cellular chromatin [21]. During reactivation, chromatin is removed to facilitate viral gene expression. However, under conditions of productive infection, viral chromatin in the form of canonical nucleosomes does not form on the viral DNA. Particularly after the onset of DNA replication, viral DNA is highly accessible and deficient in nucleosomes [22,23,24,25,26] or is associated with unstable nucleoprotein complexes [27,28].

**Figure 1:**
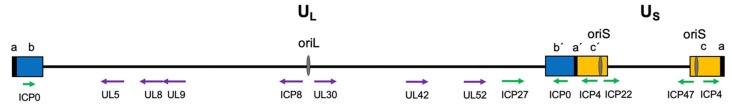
Diagram of the HSV-1 genome. The UL region is flanked by b and b’ (blue) inverted repeats and the US region is flanked by c’ and c (yellow). The three origins of replication (one OriL and two OriS) are designated as gray ovals. The genes that code for the seven core viral replication proteins are within the UL region (purple). IE genes that code for the IE proteins (ICP4, ICP0, ICP27, ICP22, and ICP44) are labeled green. Adapted from Lehman and Boehmer, Journal of Biological Chemistry, 1999 [33].

The HSV-1 genome is separated into two distinct components known as the unique long (UL) and unique short (US) regions (Figure 1). These sequences are flanked by inverted repeat sequences [29]. Within these regions are three origins of replication: OriL and two copies of OriS. OriL is located within the UL region, whereas the two copies of OriS are found in the inverted repeat short/terminal repeat short (IRS/TRS) region of the viral genome [30]. The origins contain palindromic sequences, which enable base pairing and hairpin formation. The viral origins of replication are flanked by the promoter regions of key viral genes. For instance, OriL is flanked by the genes for ICP8 and UL30 [29]. OriS is located between the genes for the immediate early proteins ICP4 and ICP22/47. Although viral genome replication can amplify viral gene expression, expression of the origin flanking genes is unaffected by the presence or absence of either OriS or OriL [31]. OriL is important for pathogenesis in mice, while any one origin is sufficient for viral DNA replication in cell culture [32].

### 2.2. HSV-1 Replication Proteins

**Figure 2:**
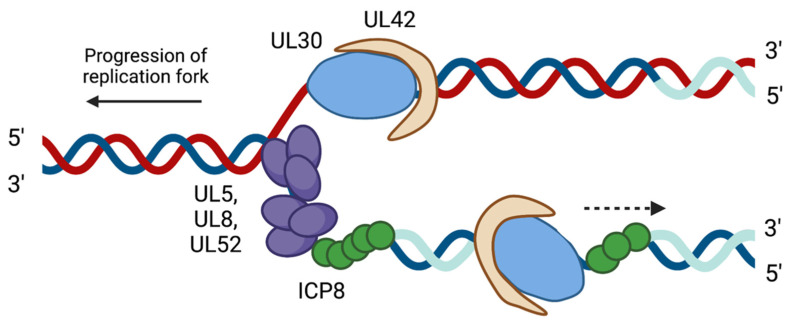
Diagram of the viral factors associated at a viral replication fork during HSV-1 DNA replication. Following the binding of the origin binding protein UL9 (not shown), DNA is unwound and RNA primers are synthesized by the helicase–primase complex (UL5, UL8, and UL52), shown in purple. The single-stranded DNA binding protein ICP8 coats single-stranded DNA (green). The viral polymerase UL30 (blue) and the processivity factor UL42 (beige) carry out leading (top) and lagging (bottom) strand synthesis. The RNA primers are shown in light blue. Created with BioRender.com, accessed on 28 September 2021. Adapted from Dembowski, Dremel, and DeLuca, PLoS Pathogens, 2017 [15].

Seven viral proteins serve as the core replication machinery. These include the origin binding protein (UL9), single-stranded DNA binding protein (ICP8), helicase–primase complex (UL5, UL8, and UL52), DNA polymerase (UL30), and processivity factor (UL42) (Figure 2). Both OriL and OriS serve as binding sites for the origin binding protein UL9, which functions to initiate viral DNA replication. UL9 has intrinsic ATPase and helicase activity that function to unwind the origins of DNA replication [34,35,36,37,38]. ICP8 in complex with UL9 binds to the single-stranded DNA preferentially in a non-sequence-dependent manner. Like other replicative single-stranded DNA binding proteins, ICP8 forms filaments and binds cooperatively to single-stranded DNA. In addition, ICP8 has strand annealing activity, which may indicate a role in viral DNA recombination [39,40]. ICP8 stimulates the viral DNA polymerase and helicase–primase complex and interacts with other viral and cellular proteins to promote viral DNA replication [41,42,43,44,45,46].

Following the interaction between UL9 and ICP8, the helicase–primase complex is recruited to viral replication forks. The helicase–primase complex is a heterotrimeric complex consisting of UL5 (helicase), UL8 (accessory protein), and UL52 (primase) [47,48]. This complex has 5’ to 3’ helicase, ATPase, primase, and DNA binding activities. Once the helicase–primase complex is recruited to the viral replication forks, UL5 further unwinds the double-stranded DNA. UL52 then adds short RNA primers to provide a substrate for DNA synthesis [49,50,51,52]. Although UL5 and UL52 have both helicase and primase activities, the third subunit, UL8, is necessary for nuclear localization of the helicase–primase complex and aids in unwinding duplexed DNA [43]. Thus, the complex works as a heterotrimer for efficient DNA replication.

Next, the viral DNA polymerase UL30 and processivity factor UL42 are recruited to viral DNA. Recruitment may be facilitated in part by an interaction between the enzymatic site of UL30 and the *C*-terminus of UL8 [53,54]. UL30 is member of the B family of DNA polymerases and is a multifunctional enzyme. UL30 has polymerase activity to extend DNA and 3’–5’ exonuclease activity to remove mismatched nucleotides [55,56]. UL30 has an approximate elongation rate of 30 nucleotides per second [15,57,58]. The slow elongation rate may help increase polymerase fidelity so that mis-incorporated nucleotides can be excised by the 3’–5’ exonuclease activity. Despite exonuclease and proofreading functions, UL30 has the potential to insert mismatches as frequently as 1 in every 300 nucleotides [57]. This may indicate that repair mechanisms could be required to help in viral genome fidelity maintenance. It is likely that HSV-1 leading and lagging strand synthesis are coordinated and the trombone mechanism of replication is supported by electron microscopy studies of in vitro replication intermediates [59].

The UL42 processivity factor plays an important role in assisting with DNA synthesis by increasing the affinity of UL30 for viral DNA. UL42 forms a heterodimer with UL30 by interacting with the polymerase catalytic subunit [60,61,62]. UL42 has a high affinity for double-stranded DNA while binding non-preferentially [63]. This sequence-independent binding is attributed to interactions between the positively charged basic amino acids of UL42 and the negatively charged phosphodiester backbone of DNA [64]. However, unlike other processivity factors, such as cellular proliferating cell nuclear antigen (PCNA), UL42 acts as a monomer and does not form a multimeric ring around the DNA. The intrinsic ability of UL42 to bind to DNA is crucial for HSV-1 replication [65]. UL42 functions to prevent the premature dissociation of UL30 from DNA by decreasing the dissociation rate of UL30 by 10-fold, while having no effect on the association rate of UL30 with viral DNA [58].

Several additional viral auxiliary factors help to facilitate viral DNA replication. These include the essential factor alkaline nuclease (UL12), which possesses 5′ to 3′ exonuclease activity [66]. In addition, non-essential viral auxiliary factors include the viral thymidine kinase (TK, UL23), ribonucleotide reductase (UL39/UL40), uracil glycosylase (UL2), and deoxyuridine triphosphatase (UL50) [67]. TK phosphorylates thymidine, other nucleoside analogs, and antivirals such as acyclovir [68,69]. Mutation of the TK gene can therefore lead to antiviral resistance.

Overall, HSV-1 DNA replication is a highly regulated and conserved process in which the functions of viral replication proteins have been well characterized in the past. The absence of one or more viral replication factors can either slow down or completely stop viral replication. In addition, viral DNA replication is coordinated with transcription, recombination, and repair. The potential roles of viral and cellular factors in coordinated regulation are discussed below.

## 3. Replication-Coupled Processes

### 3.1. Transcription

Both HSV-1 replication and transcription occur within the host cell nucleus. HSV-1 genes are expressed through a temporal cascade. IE, E, and L viral genes are transcribed chronologically by host RNA pol II. HSV-1 codes for two transcription factors: VP16 and ICP4 [70,71,72]. The viral tegument protein, VP16, binds directly to IE gene promoters to activate IE transcription [73]. The IE gene product ICP4, the major viral transcription factor, activates the transcription of E and L genes [72]. ICP4 functions to recruit other cellular complexes to aid in transcription initiation, such as cellular TATA binding protein (TBP), TFIID, and the Mediator complex [22,74,75,76,77]. There are also three other IE gene products involved in the regulation of viral transcription: ICP0, ICP27, and ICP22. ICP0 is an E3 ubiquitin ligase that counteracts host repression of the viral genome by disrupting PML entrapment, DNA damage responses, and chromatin repression of the viral genome [78]. ICP27 modulates splicing [79], 3’ processing [80,81], and mRNA export [82], while ICP22 promotes transcription elongation of viral genes [83].

The products of E genes include the viral replication machinery and viral proteins that facilitate the replication of the viral genome and the formation of replication compartments [84]. Following viral DNA replication, L genes are transcribed [85]. L gene expression is dependent on viral DNA replication, except for leaky late genes that are amplified by viral replication. The onset of genome replication is sufficient to license the expression of L viral genes [15] and results in the binding of TBP, TAF1, and RNA Pol II to the L promoters [86]. However, the mechanism by which replication alters the viral genome to allow transcription factor binding is not known. L genes code for viral structural proteins including capsid proteins and glycoproteins. Thus, viral DNA replication plays a central role in viral gene expression and the packaging of progeny virions.

### 3.2. Recombination and Repair

Initially, viral DNA replication proceeds through an origin-primed mechanism at one or more of three origins of replication and then switches to a recombination-based mechanism [13]. Recombination begins at sites of single- and double-strand breaks. Single-strand breaks (SSBs), transcription conflicts, and replication fork collisions encountered during viral DNA synthesis would be expected to cause replication fork collapse, providing substrates for DNA recombination [87]. Collapsed replication forks may result in the formation of DNA double-strand breaks (DSBs), and DSBs have been shown to trigger HSV-1 genome isomerization [88]. Intragenomic recombination occurs between the inverted repeats of the HSV-1 genome. This results in four isomers of the viral genome in equimolar amounts, depending on the orientation of the UL and US regions [89,90]. The sequences at the end of the inverted repeats (a region) undergo recombination most frequently [18]. The process of creating viral genome isomers depends on HSV-1 DNA replication and the HSV replication machinery [91]. Intergenomic recombination also occurs between coinfecting HSV-1 genomes and may contribute to the diversity of HSV-1 populations [90,92,93,94].

During viral replication, there is an accumulation of branched DNA structures [95]. Intracellular HSV-1 DNA produces Y junctions and X junctions, as visualized by both gel electrophoresis and electron microscopy [18,96,97,98,99,100]. The Y junctions are characteristic of replication forks, whereas X junctions resemble replication forks or recombination intermediates. Although cellular factors may be involved in viral recombination, HSV-1 encodes a two-protein recombinase, the alkaline nuclease UL12 and the single-stranded DNA binding protein ICP8 [101]. UL12 and ICP8 work together to catalyze a reaction that results in strand-exchange similar to that of the λ phage Red recombination system [102]. UL12 is functionally similar to the λ exonuclease, Exo, and ICP8 is analogous to the λ single-strand annealing protein, β. Similar to the λ Exo protein, UL12 removes nucleotides in a 5’ to 3’ manner [103]. It is speculated that a single-stranded tail is exposed and then annealed by ICP8 to another homologous single-stranded region [39,104]. It is not known if UL12 and ICP8 are able to function independently to cause HSV-1 DNA recombination, or if they work with the cell’s recombination machinery [101].

### 3.3. Genome Packaging into Capsids

For some population of replicated HSV-1 DNA, packaging of nascent genomes into capsids occurs in as little as 1 h after viral DNA replication [15]. This indicates that after DNA replication, there may be a segregation of function, where some genomes participate in ongoing replication and transcription, while another population is packaged. In addition, there may be unexpected interactions between the replication and packaging machinery.

## 4. Viral Replication Fork Dynamics of Cellular Factors

**Figure 3:**
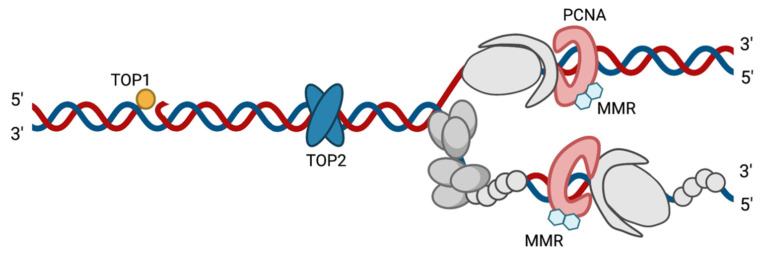
Schematic of cellular factors associated at HSV-1 replication forks. Viral replication proteins are in gray. Cellular factors found at viral replication forks that are depicted in this figure include TOP1 (yellow), TOP2 (blue), PCNA (red), and mismatch repair proteins (MMR) (light blue). Created with BioRender.com, accessed on 28 September 2021. Adapted from Dembowski, Dremel, and DeLuca PLoS Pathogens 2017 [15].

**Figure 4:**
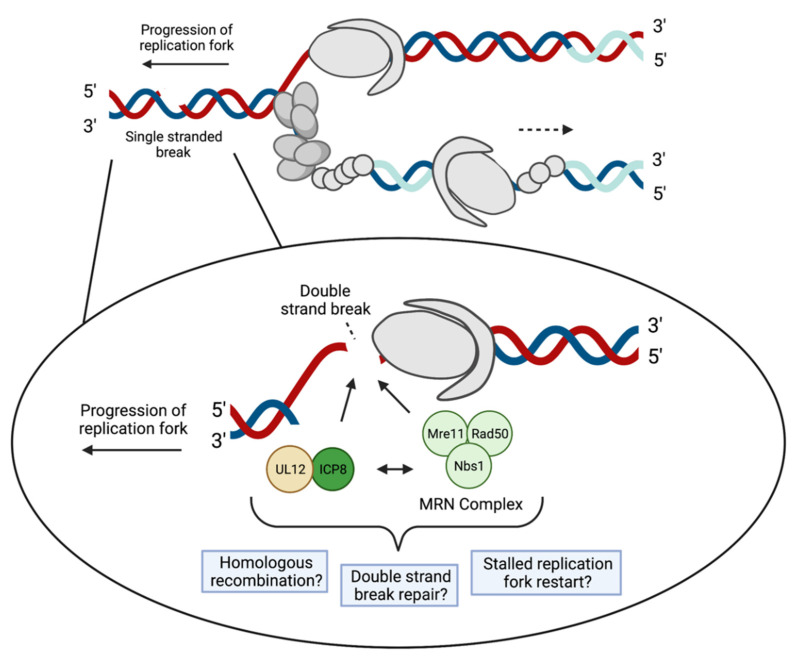
Potential role of the MRN complex in HSV-1 DNA replication. As the HSV-1 replication fork progresses, the viral polymerase UL30 may encounter single-strand breaks and gaps on the DNA. UL30 is then unable to continue synthesizing the new DNA strand, resulting in a double-strand break. Perhaps the MRN complex (light green) and the two subunit recombinase—UL12 (yellow) and ICP8 (dark green)—function together for double-strand break repair via homologous recombination to enable stalled replication fork restart. Inside the circle is a zoomed in image of what may occur at a single-strand break as the replication fork progresses on the leading strand. Created with BioRender.com, accessed on 28 September 2021.

**Figure 5:**
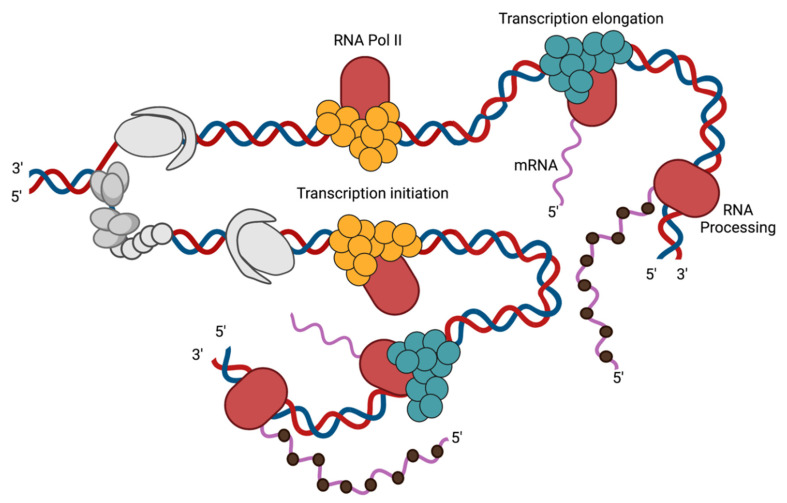
Representation of replication-dependent licensing of late gene transcription on HSV-1 DNA. Transcription initiation likely occurs directly after the replication fork. This is followed by transcription elongation and RNA processing. RNA pol II is shown in red, transcription initiation factors in orange, transcription elongation factors in blue, and RNA processing factors in black. Viral replication factors are in gray. RNA processing factors likely associate with the transcribing polymerase through interactions with the *C*-terminal tail of RNA pol II. To simplify this figure, the Okazaki fragments on the lagging strand have been removed, as well as transcription bubbles. Created with BioRender.com, accessed on 28 September 2021.

Evidence suggests that HSV-1 can manipulate cellular factors to aid in DNA replication and other replication-coupled processes. Many cellular factors are known to colocalize with viral replication compartments. However, the precise roles these factors play during infection are not well defined. The development of the isolation of proteins on nascent DNA (iPOND) technique has enabled the investigation of protein interactions with cellular and viral replication forks [15,105]. A select subset of cellular proteins have been found to be associated with replicating viral DNA [14,16] and some are specifically enriched at viral replication forks (Figure 3, Figure 4 and Figure 5) [15]. These proteins carry out a range of functions, including DNA repair, recombination, chromatin modification, transcription, and regulation of transcription-coupled RNA processing. Some cellular factors that are enriched on HSV-1 genomes during viral replication include PCNA, mismatch repair (MMR) proteins, topoisomerases (Figure 3), MRN complex proteins (Mre11 and Rad50, Nbs1) (Figure 4), and transcription factors (Figure 5) [15]. Each of these cellular proteins have the potential to play integral roles in the HSV-1 lifecycle. Although we will focus on proteins that have been found to physically interact with the viral genome, additional cellular factors are likely to be important for viral replication, which have been discussed elsewhere [91].

### 4.1. PCNA and MMR Proteins

Experiments investigating the host proteins found on replicating viral DNA have demonstrated that PCNA is one of the most enriched proteins associated with HSV-1 replication forks (Figure 3) [15]. In addition, the DNA-dependent ATPase that loads PCNA onto DNA (replication factor C composed of RFC1-5) also associates with viral replication forks, suggesting a mechanism for PCNA loading onto viral DNA. In cells, PCNA is a homotrimer that encircles DNA and functions to add processivity to cellular DNA polymerases while acting as a scaffold for various DNA damage, repair, and chromatin remodeling proteins [106]. It also plays a role in Okazaki fragment maturation and trans-lesion synthesis. PCNA is therefore important for orchestrating many processes during cellular DNA synthesis.

During HSV-1 infection, PCNA knockdown by siRNA results in reduced viral DNA replication [107]. In a separate study, when viral DNA replication was inhibited with acyclovir, there was a decrease in PCNA recruitment to HSV-1 DNA [15]. The viral neurovirulence factor ICP34.5 interacts with UL30, UL42, and PCNA in in vitro studies [108], suggesting that PCNA can either directly or indirectly associate with viral replication proteins [108,109]. Although PCNA is required for efficient HSV-1 DNA replication, its involvement is independent of its ability to complex with ICP34.5 [107]. How PCNA acts with viral replication proteins during viral DNA synthesis is not known.

Although HSV-1 has its own processivity factor (UL42), PCNA may contribute unique functions to viral DNA replication that UL42 is unable to facilitate. During cellular replication, PCNA acts as a scaffold to tether DNA repair and DNA damage response proteins to cellular DNA in the incidence of a lesion. Perhaps PCNA plays the same role in viral DNA repair, recruiting repair polymerases or proteins to viral genomes to bypass or repair DNA damage encountered or produced during replication.

PCNA-interacting proteins, including MMR proteins, were also among the most enriched cellular proteins on viral replication forks [15]. By immunofluorescence, MMR proteins (MLH1, MSH2, and MSH6) were found to colocalize with replicating viral DNA (Figure 3) [15,110]. MMR proteins are tethered to cellular replication forks by PCNA [111]. Therefore, one potential function of PCNA during HSV-1 infection may be to recruit MMR factors to viral DNA during replication. Furthermore, MSH6 physically interacts with ICP8 by coimmunoprecipitation, and both MSH2 and MLH1 are required for efficient HSV-1 gene expression and DNA replication [91,110]. During cellular DNA replication, MMR proteins are recruited by cellular PCNA to recognize base pair mismatches, insertions, and deletion mutations [112,113,114,115,116]. MMR proteins can also stimulate a DDR mediated by ATR signaling [117]. ATR and downstream targets in the Fanconi anemia pathway are important for efficient viral replication [118,119]. Perhaps PCNA tethers MMR proteins to viral replication forks to maintain viral genome integrity or to regulate the DDR.

### 4.2. Topoisomerases

Cellular topoisomerases Top1, Top2A, and Top2B are also associated with viral DNA (Figure 3). Top2A and Top2B are Type II topoisomerases and Top1 is a Type IB topoisomerase. Type II topoisomerases function to relax supercoiled DNA in front of replication forks and downstream from RNA polymerases during transcription by creating transient DSBs to allow double-stranded DNA passage. Type I topoisomerases cleave a single strand of DNA, allowing for arrangement/spatial changes during transcription and replication. Top2A and Top2B copurify with HSV-1 DNA after the onset of viral DNA replication [77], and Top2 has been shown to cleave viral DNA in a replication-dependent manner [120]. Furthermore, Top2 inhibitors block viral DNA replication and replication-dependent L gene expression [121,122]. Top1 copurifies with HSV-1 DNA throughout infection [77], and Top1 inhibitors block HSV-2 IE gene expression and downstream events in the HSV-1 life cycle [123]. These data provide evidence for mechanistic roles of cellular topoisomerases in viral gene expression and DNA replication.

### 4.3. DNA Damage Response and DSB Repair Proteins

The cellular DNA Damage Response (DDR) pathway helps to maintain genetic integrity. The DDR involves multiple cellular factors that are responsible for sensing and responding to damaged DNA. The DDR is dependent on the cell type and the type and severity of the DNA damage. Cells use several DNA damage response pathways to recognize and repair DNA damage, including base excision repair (BER), nucleotide excision repair (NER), MMR, and DSB repair. BER and NER involve the removal of incorrect bases or nucleotides from DNA. In addition, there are several cellular pathways for repairing DSBs. These include non-homologous end joining (NHEJ), homologous recombination (HR), single-strand annealing (SSA), and microhomology-mediated end joining (MMEJ) [91]. During HSV-1 infection, several DDR proteins associate with viral DNA through interactions with viral proteins or DNA lesions.

HSV-1 genomes likely undergo MMR and BER in coordination with DNA synthesis. MMR is discussed above. As evidence for BER, HSV-1 codes for the uracil glycosylase UL2, which can remove incorrect bases from double-stranded DNA. UL2, together with human AP endonuclease and the HSV-1 DNA polymerase, are capable of carrying out BER in vitro [124]. It is likely that in vivo, viral and cellular proteins act together to excise and repair mis-incorporated or damaged bases and nucleotides.

During HSV-1 infection, the mechanisms of break recognition are generally active; however, downstream steps in the DDR are inhibited through the actions of ICP0. For the NHEJ pathway, XRCC6 (Ku70) and XRCC5 (Ku80) normally recognize breaks, resulting in DNA-PK activation. During HSV-1 infection, XRCC6 and XRCC5 associate with replicating viral DNA [15]. However, the DNA-PK pathway is not active. Specifically, DNA-PK activity is reduced in infected cells as a function of ICP0 [20,125].

Cellular proteins involved in HR are also important in HSV-1 infection (Figure 4). HR is mediated by the MRN complex sensing DSBs, followed by ATM activation by the Nbs1 subunit of the MRN complex [126]. Mre11 and Rad50 associate near viral replication forks [15] and Nbs1 has been shown to colocalize with viral replication compartments [127]. Other studies have shown that during HSV-1 infection, ICP8 and UL12 interact with Rad50 and Mre11 [45,128]. Specifically, co-immunoprecipitation of ICP8 followed by mass spectrometry identified Mre11 and Rad50 [45]. By immunoprecipitation of UL12-associated proteins, a separate study demonstrated that UL12 interacts with Mre11, Rad50, and Nbs1, and that this interaction is independent of other viral proteins [128]. This may indicate that the UL12–ICP8 two-subunit recombinase may recruit the MRN complex members to the viral genome. For cellular HR, the MRN complex helps to resect the DNA at the break and carry out strand invasion. However, during HSV-1 infection, ICP0 inhibits RNF8 and RNF168 to attenuate downstream steps in HR [129]. This suggests that the MRN complex may be adapted to help facilitate a unique form of viral genome recombination in coordination with UL12 and ICP8.

In addition, MMEJ may occur on viral DNA [91]. This is a form of DSB repair that requires less homology between strands than HR. Consistently, cellular factors that mediate MMEJ, including Parp1, Xrcc1, Ligase 3, and Ercc1, all associate with replicating HSV-1 DNA [15]. MMEJ has been shown to repair DSBs at collapsed replication forks [91]. Therefore, as an alternative, or in addition to the model presented in Figure 4, MMEJ could potentially act to repair the breaks encountered during viral DNA replication.

In uninfected cells, ATR is activated in response to stalled replication forks. Further, ATM activation results in the activation of the ATR pathway. Interestingly, the ATR pathway is disrupted by HSV-1 infection despite ATM activation. The mechanism by which HSV-1 inhibits ATR signaling is through ICP8 and the helicase–primase complex. These four replication proteins bind to single-stranded DNA adjacent to double-stranded DNA. This prevents the loading of the 9-1-1 complex and TOP1, ultimately disabling ATR [118,130].

Another DDR pathway involved in HSV infection is the Fanconi anemia pathway [119]. The Fanconi pathway is activated by ATR in a response to stalled replication forks [131]. The cellular proteins involved in this pathway are important for HSV-1 replication and transcription. These proteins have been demonstrated to be regulators of DNA repair pathway choice during infection. One cellular protein, FANCI, interacts with ICP8, UL30/UL42, and UL12, whereas FANCD2 interacts with UL5 [119]. The absence of these proteins results in a decrease in HSV-1 replication. Thus, the cellular proteins in the Fanconi anemia pathway are important for HSV-1 infection.

### 4.4. Transcription Factors

Several cellular transcription factors have been found to colocalize and to copurify with actively replicating viral DNA. These proteins function in transcription regulation of cellular genes and include RNA Pol II, TFIID, the Mediator complex, LSD1, and the Integrator complex [15,132]. The TATA-binding protein (TBP)-containing complex, TFIID, and the Mediator complex are recruited to E and L viral promoters through interactions with ICP4 and help to facilitate transcription initiation [75,76,77,133,134]. A recent study showed that at the onset of viral DNA replication, RNA pol II, TBP, and TAF1 (a subunit of TFIID) bind to previously silenced L gene promoters to license L gene expression [86]. Together, these data demonstrate that viral DNA replication regulates L gene promoter accessibility to cellular transcription factors.

In pulse chase studies, factors associated with HSV-1 replication forks (pulse) were compared with factors associated with nascent viral DNA after replication (chase) (Figure 5) [15]. This study revealed that factors involved in transcription initiation, including Mediator and TFIID, were more abundantly associated with pulse-labeled replication forks. On the other hand, transcription elongation and post-transcriptional RNA processing factors, such as the Integrator complex, were relatively more abundant on nascent DNA at a distance from the replication fork. This indicates that licensing of L viral gene transcription occurs immediately after DNA synthesis. In addition, one round of replication was sufficient to license robust L gene expression [15]. Still, the mechanism by which replication alters the transcriptional competence of viral genes is not understood.

### 4.5. Chromatin Remodeling Factors

Components of the B-Wich, Swi/Snf, and NuRD chromatin/nucleosome remodeling complexes associate with viral DNA after the onset of replication [77], and the NuRD complex is enriched at viral replication forks [15]. In addition, members of the Swi/Snf, NuRD, and Ino80 complexes copurify with ICP4 from infected cells before and after the onset of viral DNA replication [135]. However, as indicated above, chromatin in the form of stable nucleosomes is not abundantly associated with viral DNA during lytic replication. Therefore, it is not clear why chromatin remodeling factors are associated with replicated viral DNA. Perhaps they have an affinity for nucleosome-free DNA through interactions with ICP4 and/or have modified functions during viral infection.

## 5. Remaining Questions

There are many unanswered questions regarding how HSV-1 DNA synthesis is coupled with other processes. (1) How do the initial rounds of viral DNA replication change the transcriptional competence of viral genomes to enable replication-dependent transcription of the late class of viral genes? (2) How are nicks and gaps that are present on infecting viral DNA navigated by the viral DNA replication machinery? (3) How are mistakes that occur during viral DNA replication repaired? (4) How does HSV-1 utilize recombination for DNA synthesis? (5) Are replication and viral genome packaging into capsids coupled? (6) How does regulation of viral DNA replication contribute to reactivation from latency? (7) How does HSV-1 modify the cell to create an optimal environment for viral but not cellular DNA replication?

HSV-1 DNA replication involves a dynamic network of both viral and cellular factors. Viral and cellular factors likely play integral roles in coupling viral DNA replication with other processes, including transcription, recombination, and repair. Equipped with new innovative methods to examine viral DNA replication in vivo, we can begin to address the involvement of cellular factors in HSV-1 replication and coupled processes. Future studies can also address the potential to target the cellular proteins involved in viral DNA replication for antiviral therapy.

In addition, infection can occur more or less efficiently within individual cells within a population [136,137]. Specifically, single-cell analysis revealed that the state of the cell at the onset of infection likely influences the number of genomes that will initiate transcription and replication, and the number of progeny virions that can be produced. With that in mind, much of what was discussed in this review was studied at the population level. In the future, it would be interesting to explore how the state of the infected cell influences viral gene expression and DNA replication.

## Data Availability

Not applicable.
